# Antibody biomarker for de novo Parkinson disease: attempted validation

**DOI:** 10.1038/s41531-018-0064-2

**Published:** 2018-09-05

**Authors:** Na Feng, Scott Simanski, Kazi Islam, Linda S. Hynan, Thomas Kodadek, Dwight C. German

**Affiliations:** 1 Department of Psychiatry, UT Southwestern Medical Center, Dallas, TX USA; 2 Department of Chemistry, The Scripps Research Institute, Jupiter, FL USA; 3 Peptide & Peptoid Synthesis Core Facilities, University of Pittsburgh, Pittsburgh, PA USA; 4 Department of Clinical Science, UT Southwestern Medical Center, Dallas, TX USA

## Abstract

Parkinson disease (PD) is a progressive neurodegenerative disease with motor symptoms that result from degeneration of midbrain dopaminergic neurons. Biomarker research seeks to identify the disease during the pre-symptomatic phase, which is a time when therapeutic intervention will be most helpful. Previously, we screened a combinatorial peptoid library to search for antibodies that are present at much higher levels in the serum of PD patients than in control subjects. One such compound, called the PD2 peptoid, was 84% accurate for the identification of de novo PD when employed as the capture agent in an enzyme-linked immunosorbent assay. This peptoid recognized an IgG3 antibody, and IgG3 levels were also found to be significantly higher in PD vs. control serum. In that study we used samples from the NINDS Parkinson’s Disease Biomarker Program. The current study sought to validate that finding using serum samples from de novo and control subjects in the Parkinson’s Progression Markers Initiative study. We found no difference in levels of antibodies captured by the PD2 peptoid in the de novo PD vs. control subjects, and no difference in IgG3 serum levels in the two groups. The failure to replicate our previous study appears to be due to the lack of difference in serum IgG3 levels between the PD and control subjects in the current study.

## Blood biomarkers: validation attempt challenges previous findings

Failure to replicate previous results dashes hopes for a non-invasive biomarker of pre-symptomatic Parkinson’s disease (PD). The synthetic, peptide-like molecule PD2 had shown promise for accurately identify PD by binding to significantly higher levels of IgG3 antibody in people with early stage PD than in healthy controls. When Dwight German at the University of Texas Southwestern Medical Center, United States, and colleagues sought to validate these findings in serum samples from subjects in a larger longitudinal study of PD progression, they found no significant differences between the levels of PD2-bound IgG3 in newly diagnosed patients who had not yet begun taking medication and control subjects who did not show symptoms of PD over a 5-year period. The serum IgG3 levels were similar between the two groups indicating that high levels of IgG3 may not be indicative of early stage PD as was originally proposed.

## Introduction

Parkinson’s disease (PD) is the second most common neurodegenerative disease afflicting the elderly and is characterized by a combination of motor and non-motor features. PD is a progressive disorder affecting multiple neurotransmitter systems. Beside the motor symptoms, non-motor features include autonomic failure, urinary incontinence, hallucinations, and dementia.^1,2^ The clinical diagnosis of PD, when applied by movement disorders specialists, is of moderate-to-high accuracy.^3,4^ It is essential that an accurate diagnosis be obtained in order to enable disease identification and clinical trial design.

Patients with PD exhibit neurodegeneration in select groups of catecholaminergic neurons along with neuroinflammation, which is characterized by activated microglia and infiltrating T cells. As T cells activate B cells, which make antibodies, it has been shown that there are disease-related antibodies in the serum of PD patients.^5–7^ Immunoglobulin G (IgG) has the ability to exert both anti-inflammatory and proinflammatory effects and may play a role in the progression of the disease, and an immunotherapy target for IgG may represent an approach to slow or stop disease.^8–10^ Previous work in our laboratory found a promising antibody biomarker, which we called PD2, which binds significantly higher levels of IgG3 antibody in PD versus control subjects and was 68% accurate in identifying PD.^11^ The PD2 peptoid was 84% accurate in identifying de novo PD. This result prompted us to further investigate and validate the accuracy of the PD2 biomarker for the identification of de novo PD.

Here, we sought to test our initial findings in blood samples from the Parkinson’s Disease Biomarkers Program (PDBP) in a substantially larger sample from a well-characterized cohort of individuals followed longitudinally in the Parkinson’s Progressive Markers Initiative (PPMI). We examined serum levels of the antibody/antibodies bound by the PD2 peptoid in two groups—“Normal Control (NC)” who remained cognitively (defined by the Montreal Cognitive Assessment) and motorically normal over an ~ 5 years period, and “PD” who were recently diagnosed with PD and had evidence from DaTscan imaging of degeneration of the nigrostriatal dopamine system. In addition to testing whether we could replicate the previously reported PD2 peptoid findings, we measured serum IgG3 levels to determine whether these levels were also elevated in the PD vs. NC subjects.

## Results

We began the study with 100 PD and 100 NC serum samples from PPMI. However, because we did not have sufficient serum from two subjects to complete all of the measurements twice, we examined two groups of 99 subjects. The age of the PD group was 60.6 ± 9.1 years (mean ± SD) and NC group (60.3 ± 11.8). The sex balance for the two groups was 49% male for the PD group and 51% male for the NC group. This sample size provides over 99% power to find biomarker differences between the PD and NC groups, based upon the means and standard deviations from our previous study.^11^

**Fig. 1:**
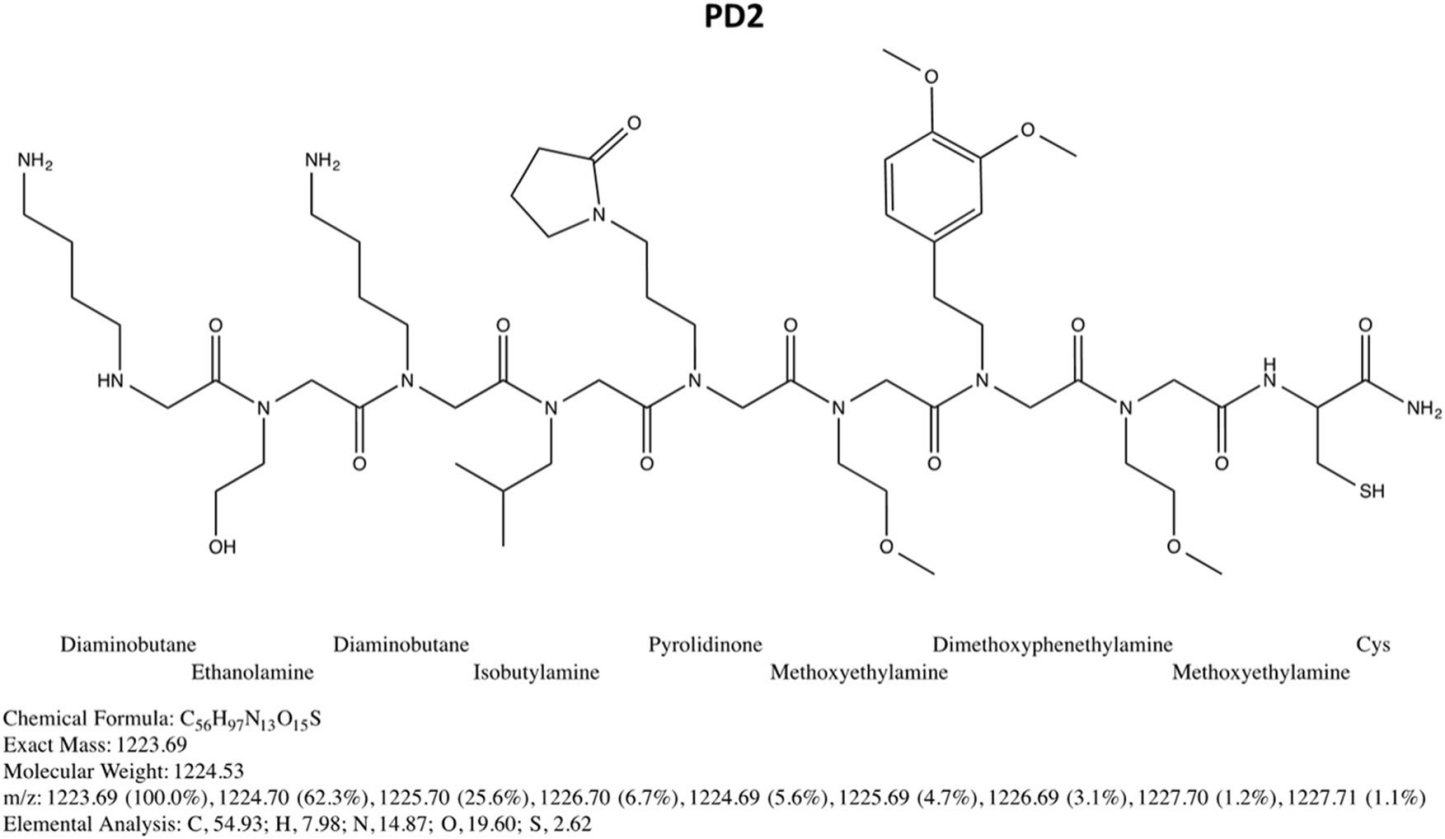
PD2 peptoid structure, illustrated with ChemDraw software

The PD2 peptoid was synthesized by a solid-phase synthesis protocol. The structure of the peptoid is shown in Fig. 1. The crude peptoid was purified by reversed-phase high performance liquid chromatography (RP-HPLC). In order to be sure that this newly synthesized peptoid performed as the one we made in our original paper (Yazdani et al.^11^), we tested the accuracy of this PD2 peptoid with serum samples used in our original study. Specifically, we performed a pilot experiment with 10 PD and 10 NC subjects which were tested previously. The 20 samples selected to test the quality of the PD2 peptoid were purposely from NC subjects with low PD2 binding and PD cases with high PD2 binding (i.e., not representative of the respective groups). The PD2 binding for these samples originally was 0.67 ± 0.15 absorbance units for the NC samples, and 2.74 ± 1.57 for the PD samples (*p* = 0.0004)). When the same samples were tested in the current study, the peptoid binding for the NC group was 1.02 ± 0.56, and for the PD group 3.17 ± 2.21 (*p* = 0.006). The current result is very similar to what we found in our original work in 2016.

**Fig. 2:**
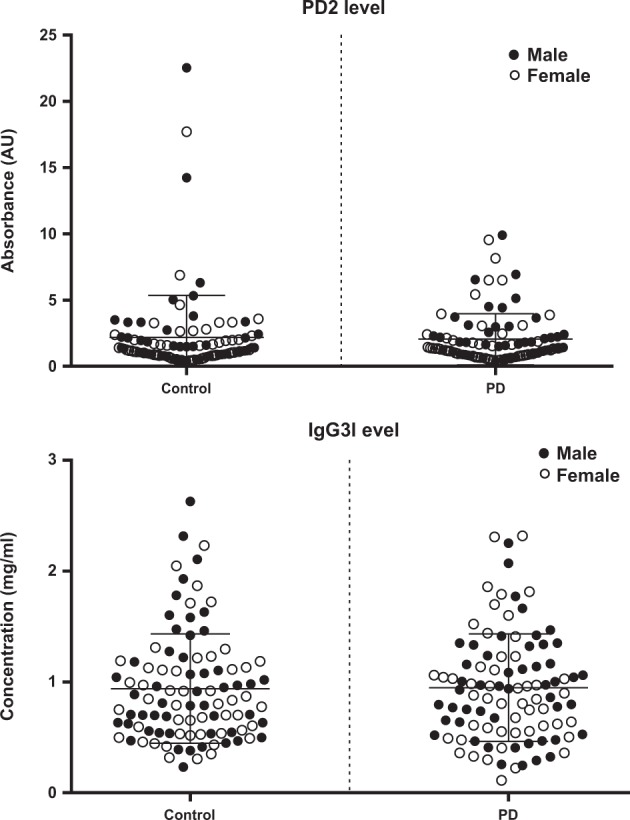
PD2 and IgG3 levels in the PPMI serum samples. Data are illustrated in absorbance units (AU). (Top) PD2-binding levels are the same for PD (*n* = 99) and control (*n* = 99) subjects. The PD2-binding levels were no different in males vs. females. Statistical data for PD2 are shown in Table 1. (Bottom) IgG3 levels are no different in PD vs. control samples. IgG3 levels are not different related to sex.

We next tested the samples from PPMI, but found no significant difference in the levels of antibodies that bound to the PD2 peptoid among the PD and NC subjects (see Fig. 2 and Table 1). We first examined the peptoid binding normalizing the serum samples based upon total serum protein levels. The result showed no difference between the PD and NC groups. As IgG is the most abundant type of antibody that could be recognized by the PD2 peptoid, we ran the samples a second time and normalized the samples based upon serum IgG levels; these are the data presented in Fig. 2. There was a highly significant correlation between the peptoid binding using both IgG and serum protein “normalization measures” (*r* = 0.79, *p* < 0.001). The failure to find a difference in peptoid binding between the NC and PD groups was not related to the sex of the subjects (*F* = 0.68), or a sex by group interaction (*F* = 0.74).

**Table 1:** Descriptive statistics for PD2 binding

Sex	Group	Mean	Std. dev.	*N*
Female	Control	1.97451	2.599005	49
	PD	1.94416	2.009070	50
Male	Control	2.38432	3.646587	50
	PD	2.15071	1.859510	49
Total	Control	2.18148	3.162229	99
	PD	2.04639	1.929394	99

In our previous study^11^ serum IgG3 levels were found to be significantly higher in the PD patients. In the present study, however, there was no difference in the levels of IgG3 between the PD and NC subjects (Table 2). Also, there was no male/female difference in the levels of IgG3 in the two groups of subjects. In our 2016 study, IgG3 levels were 47% higher in the PD vs. NC serum subjects. The mean serum IgG3 level was the same for both the PD and NC groups in the present study, and the same as that observed in the PD subjects in the previous study (i.e., 2016); however, the IgG3 levels in the NCs of the previous study was lower than in the present study.

**Table 2:** Serum IgG3 levels

	Control* (mean ± SD)	PD* (mean ± SD)	*P* value
2016 PDBP sample	0.701 ± 0.391 (*n* = 53)	1.029 ± 0.696 (*n* = 69)	0.0023
2018 PPMI sample	0.940 ± 0.492 (*n* = 99)	0.947 ± 0.485 (*n* = 99)	0.9110

PD, Parkinson’s disease. *Units: mg/ml. *P* values from Student’s independent samples *t* tests

Finally, we sought to determine whether the PD2 levels were related to the serum IgG3 levels. As analyzed by Spearman rank order correlations (rho) (Table 3), the PD2 level for all PPMI subjects (*n* = 198) was positively correlated with the serum IgG3 level (rho = 0.444, *p* < 0.001). We also analyzed the results based on PD, NC, and sex separately, and again PD2 levels were always positive correlated with the serum IgG3 levels.

**Table 3:** Spearman rank order correlation coefficients (rho) between IgG3 and PD2

	*rho*	*P value*
Overall (*n* = 198)	0.444	< 0.001
Control (*n* = 99)	0.501	< 0.001
PD (*n* = 99)	0.396	< 0.001
Female (*n* = 99)	0.555	< 0.001
Male (*n* = 99)	0.330	0.001

PD, Parkinson’s disease

## Discussion

At present, there is a great need for non-invasive and inexpensive biomarkers for neurodegenerative diseases. Progress is being made in the area of Alzheimer’s disease. Recent data suggest that exosomes in plasma can be used to identify neuropathological proteins involved with the disease,^12,13^ and panels of serum proteins have been found that can be used to identify those with the disease.^14^ Blood biomarkers for PD are being researched aggressively,^15,16^ but a useful biomarker has yet to be identified.

We identified an IgG3 antibody biomarker in the blood of PD patients that was 68% accurate for the identification of PD.^11^ The PD patients had the disease for several years and were taking medications for treatment of the symptoms. This same antibody biomarker was 84% accurate for the identification of de novo PD patients. In our 2016 study, we used subjects from the NIH Parkinson’s Disease Biomarkers Program; 75 PD patients (69 ± 5 years old), 25 de novo PD patients (62 ± 10 years old), and 104 NC subjects (69 ± 10 years old). The current study used a much larger sample of de novo patients to attempt to validate the original findings. For this validation study, 99 de novo PD and 99 NC subjects from PPMI were used, which provided > 90% power to find potential group differences. However, we found no significant difference in the level of antibody/antibodies that bound to the PD2 peptoid in the PD patients vs. NC subjects using both serum IgG level and serum total protein level to standardize the serum volume used in the enzyme-linked immunosorbent assay (ELISA).

In our previous study^11^ we found 47% higher levels of IgG3 in the serum of PD patients vs. NCs (Table 2). This was consistent with finding significantly higher levels of the IgG3 binding peptoid in both PD patients and de novo PD patients compared with NCs. In the current study, however, the serum IgG3 levels were similar in the de novo PD and NC subjects, and like that found in our original PD group. The major difference was that the current NC group was higher than the original NC group (0.94 ± 0.5 vs 0.70 ± 0.4 mg/ml). We examined the correlation between IgG3 serum level and PD2 binding and found significant positive correlations (Table 3). IgG3 levels were highly correlated with the level of PD2 peptoid-binding antibodies, which indicates that the PD2 peptoid is recognizing an IgG3 antibody in the serum, but it cannot distinguish PDs from NCs because IgG3 levels are the same in the two groups.

There are several possible reasons for the failure of the current study to validate the findings of our original study. The sample size of the de novo PD group in the original study was small (i.e., *n* = 25), which indicates that it may not be representative of the de novo population at large. We considered whether the serum collection/storage procedures differed between the PDBP and PPMI samples. However, the collection procedures are the same for both PDBP and PPMI blood sample providers, and the duration of sample storage was similar for both sample groups. Another major reason for the failure to validate our original finding is likely related to differences in the NC groups for the two studies. The IgG3 levels of the NC subjects in the PDBP samples were lower than the levels in the PPMI NC subjects (but the levels for the PD group were the same in both studies). Although the current study used de novo PD patients of a similar age to those used in the original study (~ 60 years of age̴), the ages of the NC subjects in the current study are younger than in the original study (60.3 ± 0.4, and 69 ± 5 years, respectively). IgG3 levels have been found to increase with age in men,^17^ but this finding does not correspond with the age-related change in IgG3 levels only being observed in the NC group. Clearly, the NC group from the original PDBP study was different from the NC subjects used in this validation study using PPMI samples.

## Conclusion

We have examined a relatively large sample of de novo PD and age- and sex-matched normal control subjects to validate a serum antibody biomarker for PD—the PD2 peptoid. Using samples from the PPMI we found no difference in the levels of peptoid binding to IgG3 antibodies between the two subject groups. The failure to replicate our original finding appears to be due to differences between the IgG3 levels in the NC and PD groups of the original study compared with those in the PPMI sample. The present report highlights the importance of performing large-scale replication of findings from small index studies to validate biomarkers.

## Materials and methods

### Human subjects

Serum samples were obtained from the PPMI (www.ppmi-info.org/data) on 14 February 2014. PPMI is a public–private partnership funded by the Michael J. Fox Foundation for Parkinson’s Research and funding partners (www.ppmi-info.org/fundingpartners), and all subjects gave informed consent for research utilization of their serum samples. All study protocols were reviewed and approved by the Institutional Review Board at the University of Rochester. For up-to-date information on PPMI study findings visit www.ppmi-info.org. PD patients enrolled in the study were drug-naive, had < 2-year disease duration and evidence of dopamine transporter deficit on DaTscan imaging. Subjects underwent clinical data and biospecimen collection every 3 months for the first year and every 6 months for up to 5 years. Disease severity was assessed by the Movement Disorders Society version of the Unified Parkinson’s Disease Rating Scale part III (MDS-UPDRS-III). The PD study participants had an asymmetric bradykinesia, an asymmetric resting tremor or bradykinesia, resting tremor, and/or rigidity. The subjects used in this study were selected at random and all patients met the following criteria: (a) > 30 years of age; (2) Stage of I or II for Hoehn and Yahr; (3) have not taken dopaminergic medications for > 60 days prior to the baseline visit; and (4) have not taken drugs that may interfere with the DaTscan.

### Serum collection and storage

The serum obtained from PPMI were thawed and aliquoted into 0.2 ml samples for ELISA test. Aliquots of serum were immediately placed upright in specimen storage box in a − 20 °C freezer for up to 6 h. Samples were then transferred to − 80 °C freezer for long-term storage.

### PD2 peptoid synthesis and purification

The PD2 peptoid was synthesized in solid-phase on a Liberty Blue Microwave Synthesizer (CEM Corporation) both at Scripps Florida, and at the Peptide & Peptoid Synthesis Facility (University of Pittsburgh Health Sciences Core Research Facilities) using methods previously described.^11^ In brief, synthesis is performed by stepwise addition of acylated amines to the solid support (Rink amide resin) starting from the carboxy terminus to the amino terminus (see Fig. 1). Activation of amines was performed by *N*,*N*′-Diisopropylcarbodiimide chemistry. At the end of the synthesis the peptoid was cleaved off the resin by the cleavage mixture of 90% trifluoroacetic acid, 5% triisopropyl silane, 5% water for 90 minutes at room temperature. The cleaved peptoid was subjected to multiple ether extractions. The crude peptoid was analyzed, characterized, and purified by RP-HPLC, (486 and 600E by Waters Corporation), and later confirmed to have the correct mass by MALDI-TOF Mass Spectroscopy (Applied Biosystems Voyager System 4157).

### IgG and IgG3 measurements

The total IgG levels of serum were measured in duplicates using an ELISA assay specific for IgG (Bethyl Laboratories, E80—104 and E101), according to the manufacturer’s instructions. The quantities of IgG3 in serum were assessed by using a commercial IgG3 Human ELISA Kit (ThermoFisher, BMS2094).

### Peptoid ELISA

The PD2 peptoid was immobilized onto maleimide-activated 96-well plates (Pierce Biotechnology, Rockford, IL, USA) using methods previously described.^11^ Plates were incubated in TMB substrate for 16 min at room temp and stopped with 2 mol/L H_2_SO_4_. Plates were read at 450 nm (CLARIOstar, BMG Labtech, USA). All samples were run in duplicate, and every plate contained pooled PD and NC serum samples to serve as internal controls. Results for individual samples were assessed as ratios to the NC serum pool so as to control for plate-to-plate variation. One set of ELISA assays (Scripps FL) equilibrated the serum samples according to the total serum protein level (Therno Nanodrop 3300, USA), and a second set of ELISA assays (UT Southwestern) equilibrated the serum samples according the total serum IgG levels (Bethyl Laboratories, E80—104 and E101).

### Statistics

Statistical analyses were performed using IBM SPSS V25 (IBM Corp. Released 2017. IBM SPSS Statistics for Windows, Version 25.0. Armonk, NY: IBM Corp.). The mean values of untransformed ELISA data for individual samples were compared using Students independent samples *t* test or and Mann–Whitney *U* tests as appropriate. A two-way analysis of variance was performed to examine IgG3 and PD2 levels for the two between effects of sex and diagnosis (control vs. PD). Pearson product moment (r) and Spearman rank order correlations (rho) were used to examine the association between IgG, IgG3, and PD2 for all cases, separately by sex, separately by diagnosis, and separately for diagnosis and sex combinations. PD2 levels measured from the two labs (Scripps Florida vs. UT Southwestern) were compared using Pearson product moment correlations. Statistical assumptions for all analyses were reviewed and alternative analyses were conducted if needed. Significance was set at *p* < 0.05, two-sided.

## Data Availability

The data that support the findings of this study are available from the corresponding author upon reasonable request.
